# Molecular Modeling and Structural Stability of Wild-Type and Mutant CYP51 from *Leishmania major*: In Vitro and In Silico Analysis of a Laboratory Strain

**DOI:** 10.3390/molecules23030696

**Published:** 2018-03-19

**Authors:** Masoud Keighobadi, Saeed Emami, Milad Lagzian, Mahdi Fakhar, Alireza Rafiei, Reza Valadan

**Affiliations:** 1 Pharmaceutical Sciences Research Center, Student Research Committee, Faculty of Pharmacy, Mazandaran University of Medical Sciences, Sari 48157-33971, Iran; keighobadi216@yahoo.com; 2 Department of Medicinal Chemistry and Pharmaceutical Sciences Research Center, Faculty of Pharmacy, Mazandaran University of Medical Sciences, Sari, 48157-33971, Iran; 3 Department of Biology, Faculty of Science, University of Sistan and Baluchestan, Zahedan 98168-76578, Iran; m.lagzian@science.usb.ac.ir; 4 Department of Parasitology, Faculty of Medicine, Mazandaran University of Medical Sciences, Sari 48157-33971, Iran; mahdif53@yahoo.com; 5 Molecular and Cell Biology Research Center (MCBRC), Faculty of Medicine, Mazandaran University of Medical Sciences, Sari 48157-33971, Iran; rafiei1710@gmail.com; 6 Department of Immunology, Faculty of Medicine, Mazandaran University of Medical Sciences, Sari 48157-33971, Iran

**Keywords:** *Leishmania major*, lanosterol 14α-demethylase, mutation, homology modeling, molecular dynamics, protein stability

## Abstract

Cutaneous leishmaniasis is a neglected tropical disease and a major public health in the most countries. *Leishmania major* is the most common cause of cutaneous leishmaniasis. In the *Leishmania* parasites, sterol 14α-demethylase (CYP51), which is involved in the biosynthesis of sterols, has been identified as an attractive target for development of new therapeutic agents. In this study, the sequence and structure of CYP51 in a laboratory strain (MRHO/IR/75/ER) of *L. major* were determined and compared to the wild-type strain. The results showed 19 mutations including seven non-synonymous and 12 synonymous ones in the CYP51 sequence of strain MRHO/IR/75/ER. Importantly, an arginine to lysine substitution at position of 474 resulted in destabilization of CYP51 (ΔΔG = 1.17 kcal/mol) in the laboratory strain; however, when the overall effects of all substitutions were evaluated by 100 ns molecular dynamics simulation, the final structure did not show any significant changes (*p*-value < 0.05) in stability parameter of the strain MRHO/IR/75/ER compared to the wild-type protein. The energy level for the CYP51 of wild-type and MRHO/IR/75/ER strain were −40,027.1 and −39,706.48 Kcal/mol respectively. The overall Root-mean-square deviation (RMSD) deviation between two proteins was less than 1 Å throughout the simulation and Root-mean-square fluctuation (RMSF) plot also showed no substantial differences between amino acids fluctuation of the both protein. The results also showed that, these mutations were located on the protein periphery that neither interferes with protein folding nor with substrate/inhibitor binding. Therefore, *L. major* strain MRHO/IR/75/ER is suggested as a suitable laboratory model for studying biological role of CYP51 and inhibitory effects of sterol 14α-demethylase inhibitors.

## 1. Introduction

Leishmaniasis is a major health problem in most countries that endangers more than 350 million people in the endemic area. The disease is classified as a neglected tropical disease (NTD) that is a vector-borne disease transmitted by phlebotomus sandflies [1,2]. More than 20 *Leishmania* species have been identified and depending on the causative species; the disease occurs in four forms in humans, including cutaneous leishmaniasis (CL), mucocutaneous leishmaniasis (MCL), diffused cutaneous leishmaniasis (DCL), and visceral leishmaniasis (VL) [3]. *Leishmania major* (*L. major*) the main cause of cutaneous leishmaniasis, is a major public health problem in the African and the Eastern Mediterranean regions. Globally, CL is four times more prevalent than the visceral form [4] however, the dermal form is very common in Iran, where about 37,000 cases were reported in 2012–2013. This represents an incidence of 25/100,000 people in a country where 72% of the population lived in endemic areas [1].

Lanosterol 14α-demethylase (CYP51) is a cytochrome P450 (CYP) monooxygenase involved in sterol biosynthesis that catalyzes the removal of the 14α-methyl group from different sterol precursors. This demethylation reaction is a key step in the sterol biosynthesis pathway leading to formation of cholesterol in mammals and ergosterol and ergosterol-like sterols in fungi, plants and protozoa [5]. Therefore, CYP51 has been considered a suitable target for anti-leishmanial agents since mammalian cells contain cholesterol in the cell membrane while parasite cells use ergosterol in their cell membrane. *Leishmania* CYP51 is an example of a natural plant-like sterol 14α-demethylase that can be selectively targeted for development of novel anti-protozoan compounds [6]. Cell membrane sterols are essential cellular components that contribute to the formation of functional cell membranes. Inhibition of sterol 14α-demethylase activity blocks sterol biosynthesis, which is lethal in the affected organism. Indeed, CYP51 inhibitors have been well-known as herbicides in agriculture and fungicides for the control of fungal infections in humans and food industry [7]. However, due to their potential effects on the ergosterol biosynthesis, they have been suggested for the treatment of protozoan infections such as *Trypanosoma* spp. and *Leishmania* spp. parasites [6,7,8].

Imidazoles and triazoles are two important CYP51 inhibitors that coordinate to the heme in the structure of CYP51 and inhibit the enzyme by preventing substrate binding and metabolism. Due to high demands for novel CYP51 inhibitors, some azole derivatives are being synthesized and tested on *Leishmania*. Besides azoles, lanosterol analogs, another group of CYP51 inhibitors, can be potentially used in the treatment of *Leishmania* parasites [9]. In order to investigate the potential antileishmanial effects of existing or novel CYP51 inhibitors, the compound should be, firstly, tested on the laboratory strains of *Leishmania*. However, the laboratory adapted strains of *Leishmania* may carry some genomic mutations in the sequence of CYP51 compare to wild-type strains due to successive in vitro passages. Therefore, the structure of CYP51 and efficacy of the CYP51 inhibitors would be different between the wild-type and laboratory strains. In this study, we aimed to analyze genomic sequence of CYP51 in a wild-type and a laboratory strain (MRHO/IR/75/ER) of *L. major* and then to evaluate the potential effects of probable mutations on the structure and stability of CYP51 in the both strains by computational modeling and molecular dynamics simulation. The combination of these techniques has been widely used with high level of accuracy in studying the effect of ligand binding or amino acid substitutions on the structural stability and conformational changes of proteins [10,11].

## 2. Results and Discussion 

### 2.1. CYP51 Expression and Sequence Analysis

**Figure 1:**
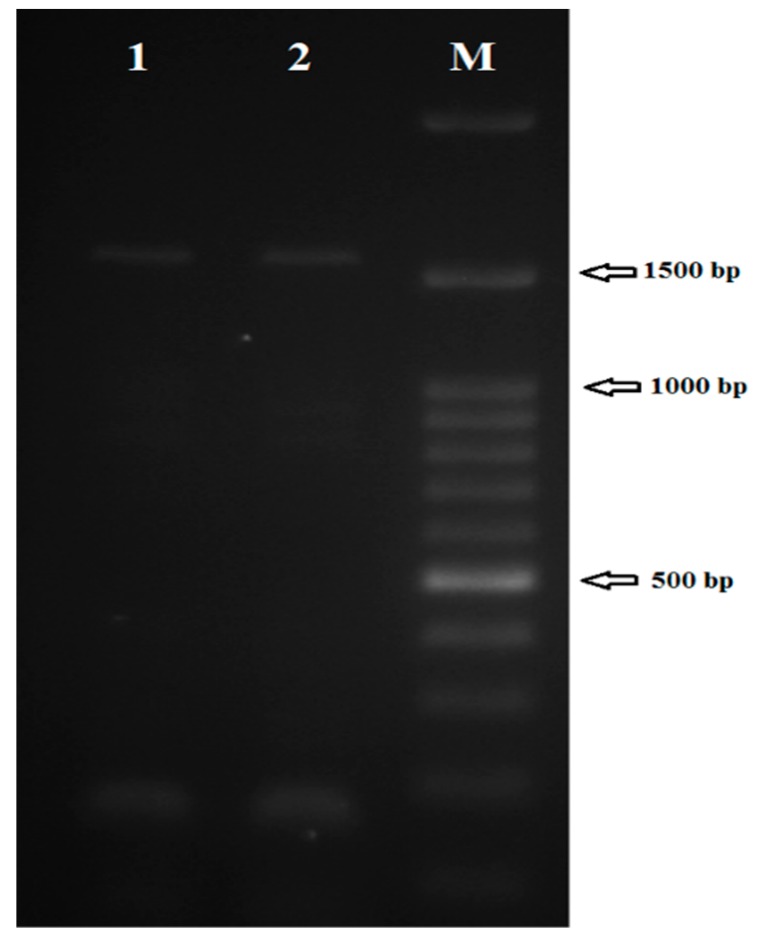
Amplification of the complete coding region of CYP51 in a wild-type (lane1) and strain MRHO/IR/75/ER of *L. major*. M: DNA ladder.

To determine whether CYP51 is expressed at the mRNA level, RT-PCR was performed. CYP51 mRNA expression was detected in an in vitro culture of *L. major* promastigotes. Products of 1538 bp were amplified in both conventional PCR and RT-PCR (Figure 1). Then, the nucleotide sequence of CYP51 of wild-type and the laboratory strain (MRHO/IR/75/ER) of *L. major* were determined. The results of DNA sequencing showed that CYP51 in *L. major* is intron-less gene consisting of 1440 bp and 480 amino acids with predicted molecular mass of 54.17 kDa for both strains. The CYP51 expression at mRNA and protein levels has already been documented for *L. infantum* and *L. donovani*, respectively [6,12]. Here we showed that putative CYP51 in *L. major* is also transcripted to mRNA; however, due to lack of available commercial anti-CYP51 antibody, we could not evaluate the expression of *L. major* CYP51 at the protein level.

**Figure 2:**
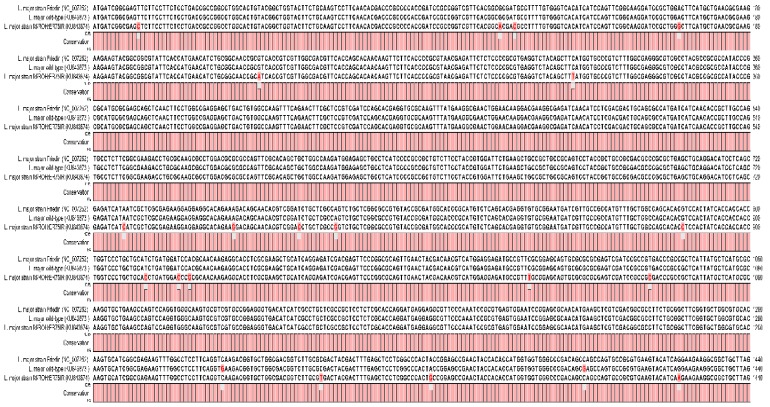
Multiple nucleotide sequence alignment of different strains of *L. major*. There are 21 nucleotide changes in these sequences, including 14 synonymous and seven non-synonymous substitutions. The nucleotide changes were shown by red letters in red boxes.

The results of DNA sequencing showed that there were some nucleotide variations in the sequence of wild-type and strain MRHO/IR/75/ER of *L. major*. Multiple alignment of CYP51 sequence, including wild-type, strain MRHO/IR/75/ER and strain Friedlin (Reference strain, accession number: NC_007252) showed that the nucleotide sequences of CYP51 in the reference and wild-type strains were almost identical except for two synonymous mutations at positions 1296 and1359; however, strain MRHO/IR/75/ER contained 19 mutations in the sequence of CYP51 that, only seven out of 19 nucleotide changes were non-synonymous substitutions leading to amino acid changes (Figure 2). The substitutions were distributed in all over the entire CYP51 sequence (Table 1).

**Figure 3:**
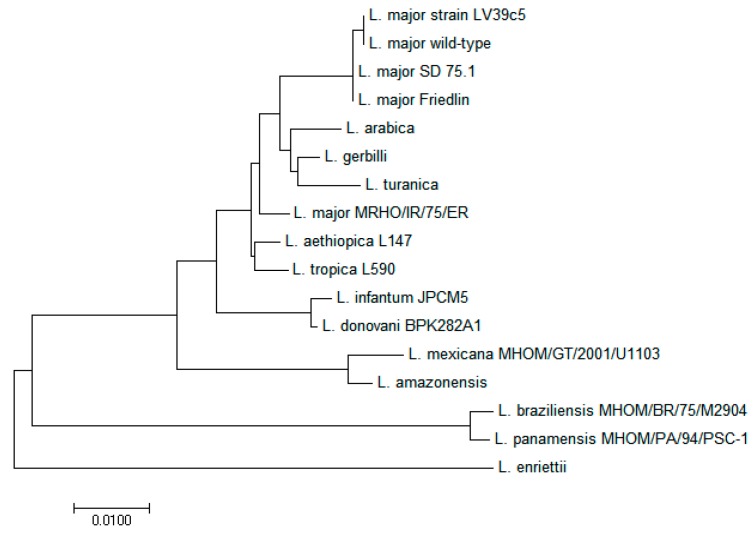
Phylogenetic analysis of CYP51 in different *Leishmania* species. Wild-type *L. major* in Iran is related to *L. major* strain LV39c5 in respect to CYP51 gene.

Whole genome sequence for 14 *Leishmania* species has been determined (https://www.ncbi.nlm.nih.gov/genome/). These include *L. major*, *L. donovani*, *L. mexicana*, *L. tropica*, *L. infantum*, *L. aethiopica*, *L. braziliensis*, *L. panamensis*, *L. enriettii*, *L. amazonensis*, *L. arabica*, *L. gerbilli*, *L. turanica* and *L. peruviana*. Putative CYP51 encoding sequences were extracted from these genomes (Supplementary Material, Table S1). The sequence of CYP51 shared high identity with other members of the *Leishmania* spp. ranging from 88 to 99 percent. Indeed, phylogenetic tree analysis of putative CYP51 in different *Leishmania* species showed that wild-type *L. major* in Iran is related to *L. major* strain LV39c5 with 100 percent sequence identity while the wild-type strain differs from *L. major* strains SD 75.1 and Friedlin in two nucleotides (Figure 3). However, there were no other CYP51 sequences for any strains of *L. major* in the database that could be used for more compassion in different regions around the world. CYP51 is well-conserved proteins that fold into structurally related domains despite some amino acid variations. The same sequence identity has been reported for other CYP-like proteins such as CYP5122A1 in *Leishmania* spp. [13] suggesting the important roles of CYPs in the biosynthesis of essential molecule and elimination of oxidative stress in *Leishmania* spp. 

**Figure 4:**
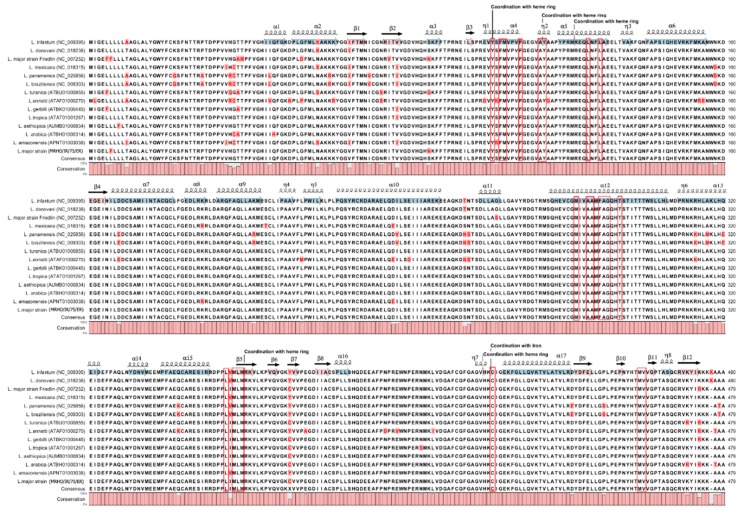
Multiple alignment of putative amino acid sequence of CYP51 in 14 *Leishmania* species. Alpha helix and beta strand were shown on the sequence. Those residues participating in the ligand binding pocket were shown with red boxes. The secondary structures were obtained from the crystal structure of CYP51 of *L. infantum* (PBD code 3L4D).

Furthermore, multiple alignment of CYP51 in different *Leishmania* species was performed to determine whether the observed amino acid changes in sequence of *L. major* strain MRHO/IR/75/ER might present naturally in the sequence of other *Leishmania* species. The results revealed that although these mutations were new in the sequence of *L. major*, these variations are naturally present in the sequence of CYP51 of other *Leishmania* spp. (Figure 4).

### 2.2. Homology Modeling and Structural Analysis of CYP51

At the protein level, the sequence identity among *Leishmania* spp. ranging from 92.69 to 100 percent identity, there is no gap within these sequences except for an extra amino acid in the sequences of *L. infantum*, *L. donavoni* and, *L. enriettii* (lysine 477). Amino acids comparison of CYP51 also revealed that those residues that are important for proper structure and function of CYP51 are conserved across these species (Figure 4). It is known that about 20 amino acids in the ligand binding pocket are conserved not only in Trypanosomatidae, including *T. brucei*, *T. cruzi* and *L. infantum* but also are conserved either across the whole CYP51 family or are phyla-specific [5,14].

Up to now, the CYP51 of *L. infantum* is the only solved crystal structure in a *Leishmania* spp. [12]. Here the 3D structure of CYP51 of wild-type and MRHO/IR/75/ER strain of *L. major* were constructed using *L. infantum* as a model (PDB entry 3L4D). The first 31 N-terminal residues of CYPY51 were not modeled in this study, as they participate in the membrane anchoring helix that folded independently from the catalytic domain [15]. CYP51 in *Leishmania* has the common P450 fold seen in other eukaryotes. Ramachandran plot analysis of the final wild-type and strain MRHO/IR/75/ER models showed that nearly all of the amino acids are in favored and allowed regions (Supplementary Material, Figure S1A). In addition, assessing the accuracy of the model by SAVES (https://services.mbi.ucla.edu/SAVES/) demonstrate it has good geometrical values. The results of ERRAT (http://services.mbi.ucla.edu/ERRAT/) software showed that the overall quality factor for wild-type and mutant models were 92.677% and 94.279%, respectively. Supplementary material, Figure S1B). These represent the percentage of the protein for which the calculated error value falls below the 95% rejection limits. As with the structure of CYP51 of *L. infantum* (template structure), the structure of CYP51 model of *L. major* revealed that the iron atom is linked to four nitrogens within the heme structure also it coordinates to Cys422, and the porphyrin ring is stabilized with hydrogen bonds with Try102, Tyr115, Arg123, Arg360 and His420 (Supplementary Material, Figure S2). 

### 2.3. In Silico Analysis of Protein Stability Upon Mutation

Next, the effect of single amino acid substitution in the structure of modeled CYP51 on the protein stability was investigated. Those residues that were found mutated in strain MRHO/IR/75/ER were selected for in silico mutagenesis studies. The in silico mutagenesis analysis was performed by replacing the wild-type residues with their corresponding mutant amino acids in the structure of wild-type modeled CYP51 of *L. major*. The results showed that all substitutions lead to decrease in the protein stability except for a serine to glycine at position of 263 (ΔΔG = −1.02 Kcal/mol) (Table 2) that resulted in stabilizing of the protein structure. Although substitutions at positions of 38, 39, 54 and 76 were destabilizing, the amount of ΔΔG between the wild-type and laboratory strain was not significant enough to change the protein stability (neutral mutation, −0.5 ≤ ΔΔG ≤ 0.5 Kcal/mol) [16]. In contrast, the arginine to lysine substitution at position 474 destabilized the CYP51 structure (ΔΔG = 1.17 Kcal/mol) in the laboratory strain, however; when the overall effects of all these substitutions were considered using molecular dynamic simulation, the final structure did not show any significant change in the protein stability. Comparing the CYP51 sequence of *Mycobacterium tuberculosis* with CYP51 sequences in other animals, plants, fungi and bacteria species, revealed that R448 (according to *Mycobacterium tuberculosis* numbering) is a highly conserved residue in the sequence of CYP51 across taxa. This residue plays an important role in folding and expression of *Mycobacterium* CYP51 but not in human and fungal orthologues. Replacement of the R448 with positively charged lysine resulted in 64% reduction in the expression of CYP51 relative to wild-type; however, the same result was not seen in human and fungal CYP substitutions. Therefore, it was suggested that despite conservation in sequence, CYP51 in different organisms can follow different folding pathways [17]. Although it has been reported that this position (474 according to *Leishmania* spp. numbering) is highly conserved in the CYP51 of different organism, this residue is not conserved in *Leishmania* species. Arginine and lysine are two amino acids that are naturally present at this position in different *Leishmania* spp. (Figure 4). Therefore, unlike *Mycobacterium*, the *Leishmania* species may use an alternative folding pathway that does not rely on arginine 474. 

A phenylalanine to leucine substitution at position of 5 in the sequence of *L. major* strain MRHO/IR/75/ER was also observed; however, it was located in the N terminal transmembrane anchoring helix of CYP51 that was not considered in the modeling study. It is believed that substitution of a hydrophobic amino acid with aromatic side chain (Phe) or to another hydrophobic but aliphatic amino acid (Leu) in the transmembarne domain can be tolerated [18]. Moreover, this variation is naturally present among various *Leishmania* species (Figure 4). Therefore, Phe/Leu substitution in the transmembrane domain of *L. major* could not affect protein stability and structure.

In another study to determine functionally and structurally important amino acids of CYP51 in rat, 45 mutations were experimentally introduced in the protein; it was shown that Y102, E347, R350 and R360 (according to *L. major* numbering) were highly conserved residues in the CYP51 that participate in the stability and expression of the protein [19]. Some other mutations in the structure of CYP51 have been reported in different fungal species. Many mutations have been reported to interfere with azole binding in *Aspergillus fumigatus* that conferred a resistance to azole compounds [20]. Importantly, a leucine to histidine substitution along with an indel polymorphism in the promoter of CYP51 has been highly associated with azole resistance in *A. fumigates* [21]. Similarly, more than 150 point mutations have been reported in the CYP51 gene of *Candida albicans* isolated from clinical samples; however, only three clusters of mutations have been linked to azole resistance suggesting the importance of mutation hotspot on the azole binding [22]. Herein all mutations were mapped at the periphery of the protein and were predicted neither to locate in the ligand binding site nor to affect structural stability.

### 2.4. Molecular Dynamics (MD) Simulations and Interpretations

**Figure 5:**
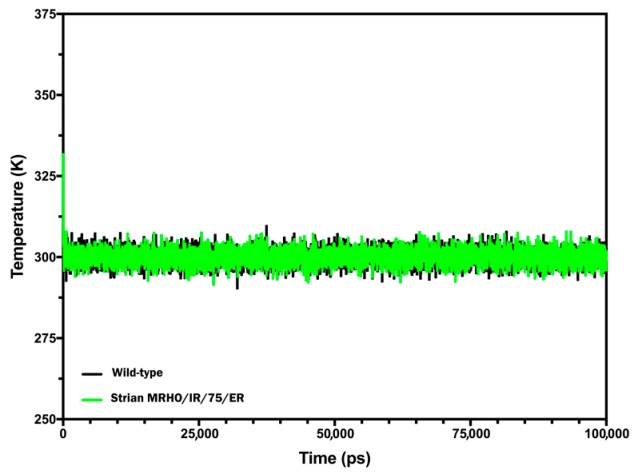
Temperature fluctuation of CYP51 protein from wild-type (black) and strain MRHO/IR/75/ER (green) during the 100 ns production step of the MD simulation.

**Figure 6:**
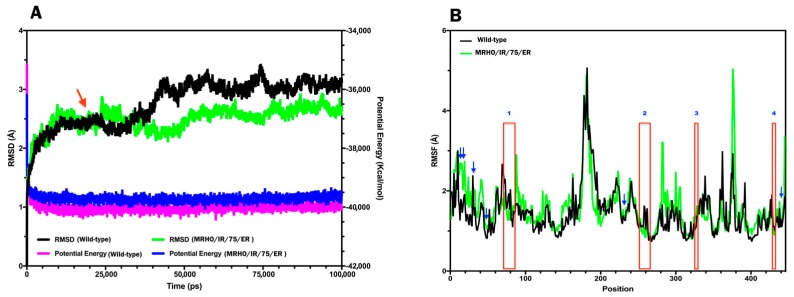
Cα RMSD and Potential energy plot of the wild-type (black and purple) and strain MRHO/IR/75/ER (green and blue) CYP51 proteins versus the simulation time. The RMSD lines were indicated by the arrow and drew relative to their starting structures (**A**). Comparative RMSF graph of 100 ns MD simulation of the wild-type (black) and strain MRHO/IR/75/ER (green) CYP51 backbone atoms. Red boxes demonstrate the approximate location of active site pocket residues. Locations of the mutations were indicated by the arrows (**B**).

To determine the stability differences between the CYP51 of wild-type and laboratory strain, MD simulation studies have been performed. The simulations were carried out for the both proteins with certain constraints as mentioned in the materials and methods section under explicit solvent conditions. The trajectory results which obtained from the 100 ns MD simulation were analyzed for Root-mean-square deviation (RMSD), Root-mean-square fluctuation (RMSF), Radius of gyration (Rg), energy terms and secondary structures. Initially, a qualitative examination on the potential energy (PE) and temperature were done to evaluate the stability of the performed simulations. The constant average fluctuation of temperature around 300 K at each step suggested the stability and accuracy of the accomplished MD simulations (Figure 5). The potential energy of the both proteins versus the simulation time showed that the average PE for CYP51 in the wild-type and laboratory strain were found around −40,027.1 and −39,706.4 Kcal/mol respectively (Figure 6A). Although, the PE for CYP51 in laboratory strain was slightly higher than wild-type strain (~300 Kcal/mol), the fluctuation of potential energy is negligible over 100 ns length of the simulation for the both of proteins, indicating that the PE reached to a stable state. As these proteins contain different number of atoms, it is not meaningful to compare the PE of both proteins and it used just for evaluating the stability of the trajectory.

The power of MD simulation on investigating proteins stability comes from comparison of conformational changes specially deviation in means of backbone dihedral angles and also the fluctuation of each residue in a protein over the course of simulation. The RMSD plots for both proteins were almost similar at the first 35 ns and difference between the RMSD of both proteins at each point were found to be less than 0.5 Å (∆RMSD is ±0.3 Å). In the next 65 ns, deviation in RMSD values a bit more increased and reached around 1 Å (Figure 6A). However, even the amount of deviation is still less than the cutoff threshold (2.5 Å) to conclude the structure of the mutant protein is changed too much. The results are supported by MD analysis of different human CYP2D6 mutations that an averaged RMSD value of 2.86 Å was seen for the wild-type and mutant proteins indicating that the structure of CYP2D6 was largely changed from that of the initial structure in comparison with the structures of mutants [23].

**Figure 7:**
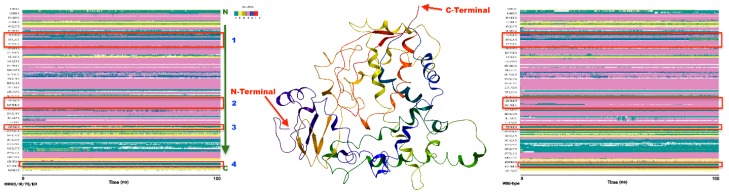
Time-base monitoring of secondary structural elements of CYP51 proteins during the simulation. For clarification, the ribbon representation of the protein with was shown in the middle of the figure. Red boxes are an approximate location of the catalytic pocket residues of the proteins. The numbers above these boxes are in accordance with Figure 6B. The color schemes of each element are as follow: T, turn; E, strand; B, isolated bridge; H, helix; G, 3_10_ helix; I, pi-helix; C, coil.

Analysis of the secondary structure content of the both proteins by the Timeline plugin of VMD 1.9.3 (http://www.ks.uiuc.edu/Research/vmd/)showed the classification of the two trajectories in terms of secondary structure elements (Figure 7). The main features of both structures are largely retained throughout the 100 ns MD. The position of residues involving in the ligand binding pocket was shown with red boxes in the Figure 7. The results revealed no significant changes between two proteins in terms of secondary structure in these regions. 

In addition, analysis of Rg value which is an indicator of protein compactness showed that globular shape of the protein is well conserved during the simulation and at the end of simulation there is only less than 4.5 Å difference between radiuses of the two proteins. Moreover, the volume of the both proteins was completely comparable to each other (76,853 Å^3^ for wild-type vs. 76,169 Å^3^ for laboratory strain). 

The analysis of RMSF showed that the average differences between fluctuations of the all residues of both proteins were less than 0.11 Å throughout the simulation. The ∆RMSF (RMSF_MT_-RMSF_WT_) for residues 7, 8, 23, 45 and 232 corresponding to the position of mutations were 0.8, 0.4, 0.5, 0.3 and 0.02 Å respectively (Figure 6B). In contrast, some other residues such as V377, L376, H283, D378, R282, K445, H13 and Y88 showed a greater structural flexibility than the average differences of fluctuation for the all residues. However, these residues which located in the non-critical regions of the protein may not affect substrate/ligand binding. RMSF analysis of residues participating in the ligand binding pocket (red boxes in the Figure 6B) showed again no differences of fluctuation between wild-type and laboratory strain. RMSF of the proteins is also another evidence of structural similarity. A RMSF plot shows the mean of fluctuation of each amino acids over the time of simulation and can be used for identification of highly flexible or unstructured region of a protein. A tyrosine to histidine (Y319H) substitution in the structure of lanosterol 14 α demethylase of *Aspergillus fumigatus* led to increase conformational flexibility (RMSF) of the protein due to change from hydrophobic interaction of tyrosine to polar interaction of histidine [24]. The t-test results for comparisons of Rg, volume of the proteins and RMSF values between wild-type and mutant proteins indicated no significant differences.

## 3. Material and Methods

### 3.1. Parasite Culture

*L. major* strain MRHO/IR/75/ER was cultured three days in RPMI1640 (Biosera, East Sussex, UK) supplemented with fetal bovine serum 10% (Biosera East Sussex, UK), penicillin (100 IU/mL) and streptomycin 100 μg/mL (Biosera East Sussex, UK). The promastigotes were grown in a cold incubator (Memmert, Schwabach, Germany) at 24 °C.

### 3.2. Genomic DNA Extraction and RNA Extraction 

Genomic DNA and total RNA were extracted from the promastigote stage of *L. major*. About 10^6^ parasites were pelleted at 1200× *g* for 10 min. The parasites were washed two times with ice-colded PBS. Genomic DNA was extracted using phenol chloroform extraction methods. Total RNA was extracted using 5 PRIME-Isol-RNALysis Reagent according to the manufacturer’s protocol. Another DNA sample was also extracted from a clinical sample of CL isolated from Turkman Sahra, as *L. major* endemic focus, in northeastern Iran. The quality and quantity of the extracted DNA and RNA were checked by agarose gel electrophoresis and spectrophotometry, respectively. The DNA was stored at −20 °C for further analysis, and the total RNA was used immediately for cDNA synthesis. 

### 3.3. cDNA Synthesis and Polymerase Chain Reaction (PCR)

Specific primers were designed to amplify the complete open reading frame (ORF) of CYP51 in *L. major*. Primer were designed by Allele ID version 7.5 (Premier Biosoft, Palo Alto, CA, USA) using *CYP51*DNA sequence (GeneID: 5649863) as a template. The forward and reverse primer sequences were as follows:

CYP51-F: 5′-TACCACCGCACACTACAT-3′

CYP51-R: 5′-ATCTCTCTCGGGTTTTCATC-3′

To detect whether *CYP51* was expressed at the mRNA level in *L. major*, 1 µg of the extracted RNA was converted into cDNA using cDNA synthesis kit (Aryatous, Tehran, Iran) according to manufacturer’s instructions, using random hexamer primers. The PCR and RT-PCR reaction were performed in total volume of 25 µL containing 10 mM Tris–HCl, pH 8.4, 50 mM KCl, 1.5 mM MgCl_2_, 250 μM of each dNTP, 20 pmol of each primer (Bioneer, Munpyeong-dong, Dae, South Korea), and 1 U *Taq*DNA polymerase (Bioneer, Munpyeong-dong, Dae, South Korea) using 2 μL of cDNA and DNA as templates. For amplification, samples were cycled in an Eppendorf thermocycler (Eppendorf Scientific, Hamburg, Germany) with an initial denaturation at 94 °C for 2 min, followed by 35 cycles of 94 °C for 30 s, 60 °C for 30 s, 72 °C for 120 s, with a final extension at 72 °C for 5 min. PCR products were analyzed by agarose gel electrophoresis (1.2%) after green viewer (Aryatous, Tehran, Iran) staining and visualized under ultraviolet transillumination. 

### 3.4. DNA Sequencing and Sequence Analysis

The specific bands according to the expected amplicon size of CYP51 were extracted from agarose gel using Gel recovery kit (Denazist Asia, Mashhad, Iran) and following the manufacturer’s protocol. The purified PCR products were eluted in distilled water and then subjected to direct sequencing with specific forward and reverse primers. The DNA sequencing was performed using capillary DNA analyzer (ABI 3730, Applied Biosystems, Foster City, CA, USA) after sequencing reactions with a Big Dye Terminator V3.1 Cycle Sequencing Kit (Applied Biosystems, Foster City, CA, USA). Forward and reverse nucleic acid sequence data were assembled to construct a continuous sequence of the target DNA, then the nucleotide sequences were analyzed using CLC Mainwork bench version 5.5 (Qiagen, Hilden, Germany). The nucleotide sequence data of CYP51 from wild type and strain MRHO/IR/75/ER of *L. major* were submitted to GenBank database under the accession number of KU843873 and KU843874, respectively. Phylogenetic tree of putative CYP51 in different *Leishmania* species (supplement Table S1) was computed using the Neighbor-Joining method [25] by MEGA7 software (https://www.megasoftware.net/) [26].

### 3.5. Homology Modeling and Sequence Analysis

The genomic sequences of CYP51 for *Leishmania* species were identified using blast search (https://blast.ncbi.nlm.nih.gov/Blast.cgi). The DNA sequence of CYP51 for *L. infantum* (gene ID: 5067288) was used as a query to identify putative CYP51 coding sequence in other *Leishmania* species. The query was blasted against reference genomic sequence (refseq_genomic) and whole-genome shotgun sequence contings (wgs) databases and those entries scoring more than 1800 were selected for further analysis. The putative CYP51 coding sequences of 14 *Leishmania* species were imported to CLC main work bench and then translated to proteins. In order to maintain data consistency and avoiding occasional output data incompatibility between different bioinformatics softwares, protein modeling simulation and analysis were performed using ICM-pro (Molsoft L.L.C. San Diego, CA, USA). These analyses included model optimization, minimization and refinement however, the final model for each protein was evaluated using SAVE sever. ICM-pro was also used for structure visualization and superimposing.

The homology modeling procedure in ICM-pro consists of the following steps: (1) The primary protein sequences of CYP51 from wild-type and strain MRHO/IR/75/ER of *L. major* were imported to the software, and PDB searched for similar and homologous structure. (2) Appropriate PDB template (Crystal structure of CYP51 from *L. infantum* PDB code: 3L4D) was selected, and the query sequence was aligned against the template primary amino acid sequence. (3) The conformational structure of the template was optimized including, deletion of water molecule, optimization of His, Pro, Asn, Gln and Cys, optimization of hydrogens and assignment of ideal geometry of residues. (4) Automated protein modeling was performed using the internal mechanics force field (ICMFF). It predicts side-chain torsion angles by simultaneous global optimization of the energy for all non-identical residues [27]. Protein side chain and loop are modeled by internal coordinate definition of the molecular object combined with computationally efficient Biased Probability Monte Carlo (BPMC) optimization [28]. (5) The modeled structure was then checked by protein health option inside ICM and finally the side chains and loops were energy minimized and refined. The final model for each structure was further evaluated with the SAVE server (https://services.mbi.ucla.edu/SAVES/) [29]. The tools that were used for evaluation include PROCHECK, WHAT_CHECK, ERRAT, VERIFY_3D, PROVE and Ramachandran Plot. The original crystal structure was also evaluated with SAVE to compare the constructed models with the crystal structure.

### 3.6. In Silico Site-Directed Mutagenesis

The effects of missense mutations identified in the sequence of the *L. major* strain MRHO/IR/75/ER on protein stability were investigated using ICM-pro. Individual mutation was introduced in the structure of the modeled CYP51 of *L. major* and the free energy change in protein stability that was computed as the difference between free energy of the unfolded and misfolded states:

∆∆G = ∆G_mutant_ − ∆G_Wt_

where:

∆G = ∆G_folded_ − ∆G_unfolded_

The free energy of the unfolded and misfolded states is approximated by a sum of the residue specific energies. The residue specific energies were derived empirically using a large set of experimental data that are implemented in the software [30]. Mutation of a given residue is followed by ICM Biased Probability Monte Carlo (BPMC) method [28] with flexible side chains for the mutated residue and its neighboring residues. The rest of the protein structure is considered rigid. A positive energy value indicates that the mutation is likely to be destabilizing.

### 3.7. Molecular Dynamics Simulation

To better understand any probable effect of the mutations on the fluctuations, conformational changes and stability of the protein, molecular dynamics (MD) simulation was carried out as follows: the simulation with implicit solvation models significantly destabilize this protein. Therefore, an explicit solvation model was used to more closely resemble the cellular environment. Thus, the protein was solvated in the TIP3 water model using CHARMm (https://www.charmm.org/) with explicit spherical boundary with harmonic restrain model. The counterion (NaCl) was added to the system at 20 mM concentration to neutralize the surface charges of the protein. The total number of water molecules added around the protein was 2103. Subsequently, the system was minimized by 500 steps of Adopted Basis NR to RMS gradient of 0.001 to clean up any Van der Waals (VDW) clashes. Then, the system was typed with CHARMm forcefield [31] and heated up to 300 K for 1 ns with 2 fs time step to pull back the structure from local minima. Nonbonded interactions cutoff was set to 12 Å and spherical cutoff method was used to treat long range electrostatics interactions. Subsequently, the system was equilibrated for 10 ns at the target temperature with already described parameters prior to the production phase. The convergence of the equilibration step was monitored by visually looking at density of solvent around the protein and monitoring energies. The production step was conducted by BIOVIA Discovery Studio 2017 using NAMD 2.9 module (BIOVIA, San Diego, CA, USA) [32]. The production time was of 100 ns and conducted on an NPT ensemble with keeping pressure and temperature constant at 1.01 bar and 300 K using Langevin Piston and Langevin Dynamics methods respectively. The SHAKE algorithm was used to constrain non-water bond. The other parameters were as previous steps. Finally, the trajectory file was analyzed for changes in root mean square deviation (RMSD), root mean square fluctuation (RMSF), Radius of Gyration (RG), secondary structures and energy terms during the simulation. These analyses were done by implemented tools in the Discovery Studio package (BIOVIA, San Diego, CA, USA). The secondary structures were analyzed by Timeline plugin of VMD software. Additionally, *t*-test was also used for statistical comparison of Rg, volume of the proteins and RMSF values between various conformations of the two proteins (*p*-value < 0.05).

## 4. Conclusions

In conclusion, we have identified seven non-synonymous mutations in the sequence of the laboratory strain (MRHO/IR/75/ER) of *L. major*. Using protein modeling and in silico mutagenesis studies, we showed that these seven mutations could not alter the protein stability and folding. Furthermore, when they were mapped on the modeled structure, they did not interfere with substrate/inhibitor binding as they were located at the protein periphery. MD simulation also confirmed the results of in silico mutagenesis studies indicating that the observed mutations in the structure of CYP51 in the laboratory strain did not affect RMSD, RMSF, RG, energy terms and secondary structure of the protein. Regarding the in silico analysis performed in this study, *L. major* strain MRHO/IR/75/ER could be used as a laboratory model for evaluation of CYP51 inhibitors in in vitro environment. However, the effects of CYP51 inhibitors on clinical samples of *L. major* should be considered separately. We also suggest that much caution should be considered when working with laboratory models of different organisms as the genomic sequences and phenotypes of wild-type and laboratory models would be different. Thus, genomic differences and their relevant effects on laboratory models should be first evaluated before working on them. 

## Figures and Tables

**Table 1:** Nucleotide and amino acid changes in the CYP51 sequence of wild-type and strain MRHO/IR/75/ER of *L. major*.

Nucleotide/Codon Position *	Substitution Type	Nucleotide and Amino Acid Changes **	Nucleotide/Codon Position *	Substitution Type	Nucleotide and Amino Acid Changes **
13/5	Nonsynonymous	T/C; Phe/Leu	915/305	Synonymous	T/C
112/38	Nonsynonymous	G/A; Ala/Thr	924/308	Synonymous	T/C
116/39	Nonsynonymous	T/C; Met/Thr	927/309	Synonymous	A/G
161/54	Nonsynonymous	A/G; Asp/Gly	1020/340	Synonymous	C/T
226/76	Nonsynonymous	G/A; Val/Ile	1053/351	Synonymous	T/C
312/104	Synonymous	C/T	1296/432 ***	Synonymous	C/G
729/243	Synonymous	A/C	1323/441	Synonymous	C/T
759/253	Synonymous	A/G	1353/451	Synonymous	A/G
777/259	Synonymous	T/C	1395/465 ***	Synonymous	C/G
787/263	Nonsynonymous	A/G; Ser/Gly	1421/474	Nonsynonymous	G/A; Arg/Lys
882/294	Synonymous	G/C			

* The positions of nucleotide were reported corresponding to the CYP51 coding region (CDS) of *L. major* strain Friedin (Accession number NC_007252). ** CYP51 coding region (CDS) of *L. major* strain Friedin (Accession number NC_007252) was used as a reference sequence and the nucleotide and amino acid changes were reported compared to it. *** These two substitutions were identified in the sequence of wild-type strain whereas the other substitutions were identified in the sequence of the strain MRHO/IR/75/ER.

**Table 2:** In silico calculation of protein stability upon mutation in the structure of *L. major* CYP51.

Amino Acid Position	Amino Acid Changes	ΔΔG (kcal/mol)
5 *	Phe/Leu	-
38	Ala/Thr	0.20
39	Met/Thr	0.50
54	Asp/Gly	0.53
76	Val/Ile	0.44
263	Ser/Gly	−1.02
474	Arg/Lys	1.17

* Not included in the final model.

## Supplementary Materials

The following are available online. Figure S1: Ramachandran plots for conformational model of CYP51 in wild-type and laboratory strain of *L. major*, Table S1: List of putative CYP51 sequences identified in different leishmania species. Figure S2: Structural model of superimposed CYP51 from *L. infantum* (PDB code: 3L4D), wild-type and strain MRHO/IR/75/ER of *L. major*.
