# Supplementary material for: Molecular Modeling and Structural Stability of Wild-Type and Mutant CYP51 from Leishmania major: In Vitro and In Silico Analysis of a Laboratory Strain
Source: Molecules. 2018 Mar 19;23(3):696. doi: 10.3390/molecules23030696 (PMC6017637; doi:10.3390/molecules23030696)
Supplement: Supplementary file 1 [file molecules-23-00696-s001.pdf]

Supplementary Material

Molecular Modeling and Structural Stability of Wild-Type and Mutant CYP51 from *Leishmania Major*: In Vitro and In Silico Analysis of a Laboratory Strain

Masoud Keighobadi [1], Saeed Emami [2,*], Milad Lagzian [3], Mahdi Fakhar [4,5], Alireza Rafiei [5,6] and Reza Valadan [5,6,*]

1 Pharmaceutical Sciences Research Center, Student Research Committee, Faculty of Pharmacy, Mazandaran University of Medical Sciences, Sari, 48157-33971, Iran; keighobadi216@yahoo.com
2 Department of Medicinal Chemistry and Pharmaceutical Sciences Research Center, Faculty of Pharmacy, Mazandaran University of Medical Sciences, Sari, 48157-33971, Iran
3 Department of Biology, Faculty of Science, University of Sistan and Baluchestan, Zahedan, 9816876578, Iran; m.lagzian@science.usb.ac.ir
4 Department of Parasitology, Faculty of Medicine, Mazandaran University of Medical Sciences, Sari, 48157-33971, Iran; mahdif53@yahoo.com
5 Molecular and Cell Biology Research Center (MCBRC), Faculty of Medicine, Mazandaran University of Medical Sciences, Sari, 48157-33971, Iran
6 Department of Immunology, Faculty of Medicine, Mazandaran University of Medical Sciences, Sari, 48157-33971, Iran; rafiei1710@gmail.com
* Correspondence: sd_emami@yahoo.com (S.E.); rvaladan@mazums.ac.ir (R.V.); Tel.: +98-113-354-3082 (S.E.); +98-911-327-9036 (R.V.)

Supplementary Table S1: List of putative CYP51 sequences identified in different *Leishmania* species.

Species	Accession number	Genomic region	CDs size (bp)
L. infantum	NC_009395	453693 to 455135	1443
L. donovani	NC_018238	456337 to 457779	1443
L. major strain Friedlin	NC_007252	464294 to 465733	1440
L. mexicana	NC_018315	442773 to 444209	1440
L. braziliensis	NC_009303	413210 to 414649	1440
L. panamensis	NC_025856	467143 to 468582	1440
L. enrietti	ATAF01000270	152371 to 153807	1440
L. tropica	ATAT01001267	68292 to 69731	1440
L. arabica	ATBH01000314	17025 to 18464	1440
L. gerbilli	ATBK01000445	95610 to 97049	1440
L. turanica	ATBU01000855	1011 to 2450	1440
L. amazonensis	APNT01003038	2608 to 4047	1440
L. aethiopica	AUMB01000834	3589 to 5028	1440
L. major strain (MRHO/IR/75/ER)	KU843873	15 t0 1454	1440
L. major (wild-type Tork-245)	KU843874	15 t0 1454	1440

A

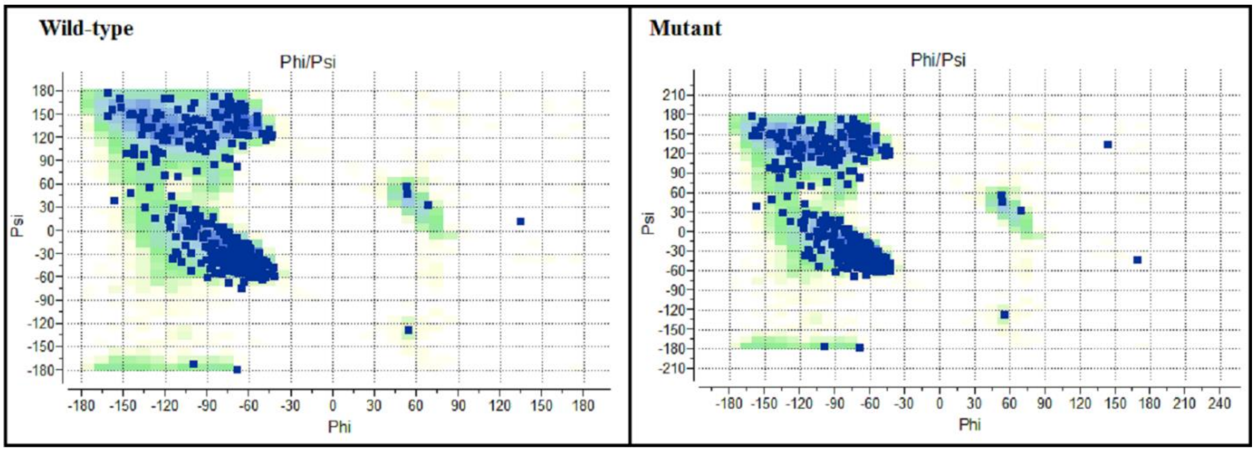

B

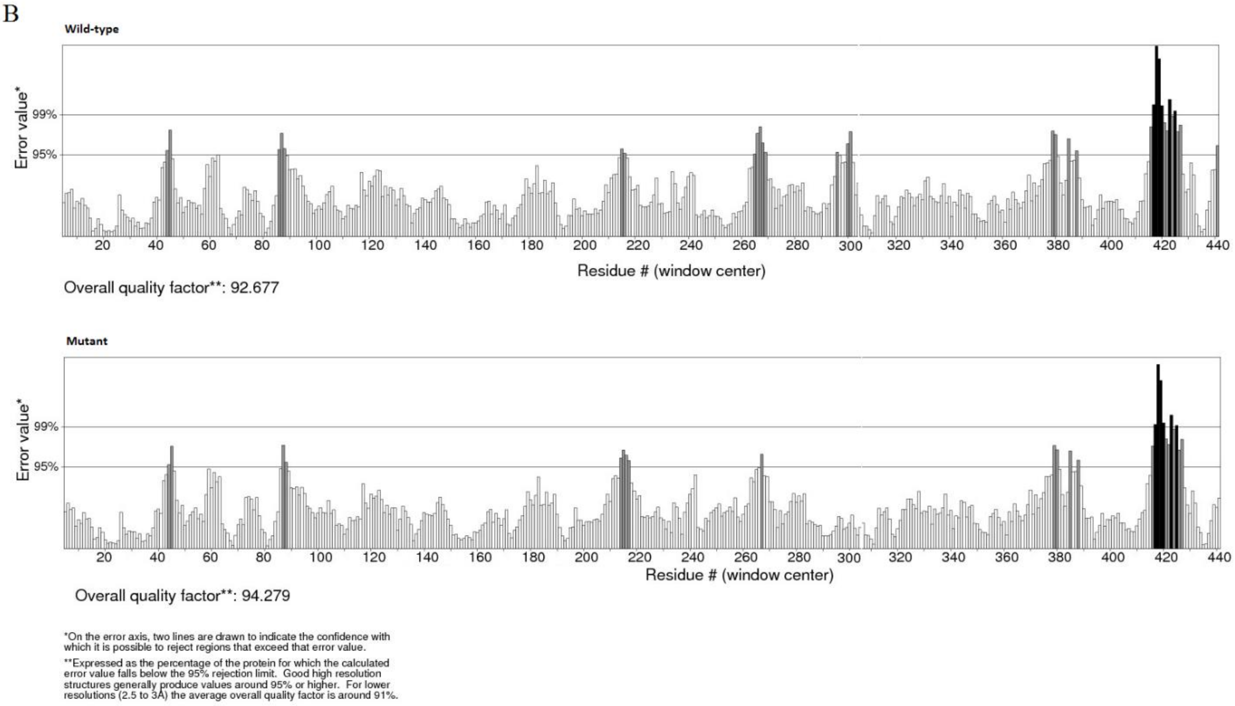

Supplementary Figure S1: Ramachandran plots for conformational model of CYP51 in wild-type and laboratory strain of *L. major.* The dark green regions contain the most favorable combinations of phi and psi while the light green regions indicate the allowed regions. The amino acids are shown with blue squares. B) ERRAT analysis (32) of wild-type and mutant structure showed that the overall quality factor for wild-type and mutant models were 92.677% and 94.279%, respectively.

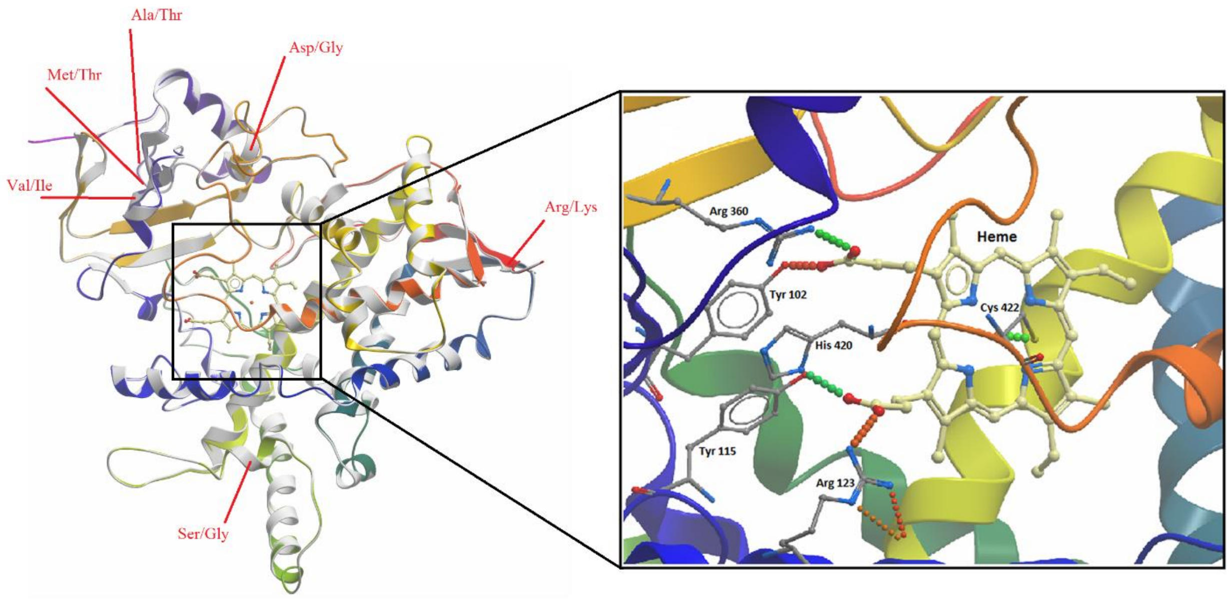

Supplementary Figure S2: Structural model of superimposed CYP51 from *L. infantum* (PDB code: 3L4D), wild-type and strain MRHO/IR/75/ER of *L. major.* (A)The three structures were superimposed. The position of non-synonymous mutations have been mapped on the structure with res lines. Different colors were used for better visualization of the secondary structures in the model (B) The porphyrin ring of heme structure was coordinated by hydrogen bonds with Try102, Tyr115, Arg123, Arg360 and His420 and the iron atom in the heme is linked to Cys422. The green and brown spheres indicates hydrogen bonds. The figure was generated by ICM-pro software.
